# Parallel behavioral and morphological divergence in fence lizards on two college campuses

**DOI:** 10.1371/journal.pone.0191800

**Published:** 2018-02-14

**Authors:** Amanda Sparkman, Stephen Howe, Stephanie Hynes, Brooke Hobbs, Karina Handal

**Affiliations:** 1 Department of Biology, Westmont College, Santa Barbara, California, United States of America; 2 Department of Biology, University of Akron, Akron, Ohio, United States of America; Uppsala Universitet, SWEDEN

## Abstract

The spread of urban development has dramatically altered natural habitats, modifying community relationships, abiotic factors, and structural features. Animal populations living in these areas must perish, emigrate, or find ways to adjust to a suite of new selective pressures. Those that successfully inhabit the urban environment may make behavioral, physiological, and/or morphological adjustments that represent either evolutionary change and/or phenotypic plasticity. We tested for effects of urbanization on antipredator behavior and associated morphology across an urban-wild gradient in the western fence lizard (*Sceloporus occidentalis*) in two California counties, Santa Barbara and San Luis Obispo. We compared college campuses in both counties with adjacent rural habitats, conducting field trials that allowed us to characterize antipredator behavior in response to the acute stress of capture. We found notable divergence between campus and rural behavior, with campus lizards more frequently exhibiting diminished escape behavior, including tonic immobility, and lower sprint speeds. Furthermore, campus females had significantly shorter limbs, and while this did not explain variation in sprint speed, those with shorter limbs were more likely to show tonic immobility. We hypothesize that these parallel behavioral and morphological changes on both campuses reflect adjustment to a novel environment involving changes in predation and human presence.

## Introduction

Across the globe, human-induced environmental change is having profound impacts on other species, through habitat loss and alteration, spread of invasive species, pollutants, and climate change [1–6]. In anthropogenically-altered habitats, species may experience changes in key biotic factors, such as predators, parasites, competitors, and prey, as well as changes in abiotic factors, such as temperature, noise, traffic, and substrate type[7–9]. Changes in all of these factors can affect the genetics, physiology, morphology and behavior of wild populations, and have critical ramifications for reproduction, survival, and ultimately population persistence [10–13].

Organisms faced with anthropogenic environmental change have three options: emigrate, perish, or adjust[12,14]. While some species are rare or absent in urban/suburban environments, others have successfully colonized the urban environment and appear to be thriving [15]. Recent work has shown diverse adjustments in physiology, behavior, and even morphology in urban habitats. For instance, some urban species have been shown to have altered stress physiology and immune function [16,17]. In birds, there is now widespread documentation of song alterations in response to urban noise [18], and a reduction in flight-initiation distance (FID) is widespread trend across species in urban environments [9]. There is also evidence of alterations in bill size in house finches, limb length in *Anolis* lizards, and wing length in cliff swallows associated with colonization of urban areas [19–21].

In some cases, organismal differences between urban and rural environments may represent a nonrandom immigrant effect (where only individuals with certain characteristics tend to colonize urban areas), plasticity, or evolutionary change in response to the urban environment itself [22,23]. Regardless of the mechanism, it has been proposed that novel, human-altered environments might favor more flexible, exploratory, bold, and or/aggressive individuals [12,24,25]. That said, in addition to being a source of novelty, complex alterations in urban environments that affect community interactions, such as predation, food availability, and retreat sites, may also shape animal personalities [22]. Much more work needs to be done to understand how different species respond to these novel selective contexts.

Studies suggest that predator communities may be significantly altered, and predation rates (though not necessarily predator numbers) reduced in urban environments [11,26,27]. This can have profound ramifications for antipredator behaviors in urban species. Where urbanization reduces the threat of predators, we would expect a relaxation of selection on antipredator behaviors, and any associated morphology and/or changes in developmentally-plastic antipredator behaviors [22,26]. In this case, we might predict diminished ability to respond adaptively to predatory threats, which may be demonstrated by reduced aggression and reduced escape behaviors. Reduced reactiveness can be beneficial in that it may free up time or energy for other important behaviors, such as foraging, courtship, thermoregulation, and other fitness-related traits [28].

The western fence lizard, *Sceloporus occidentalis* is an excellent species in which to investigate organismal response to urbanization, as past studies have demonstrated morphological, physiological, and behavioral differences between geographically distinct populations [29–32]. These lizards have successfully colonized many urban/suburban areas, and are commonly found in parks, yards, and similar areas across their distribution. Recent work in closely related eastern fence lizards, *S*. *undulatus*, has shown that they are able to make behavioral, physiological and morphological adjustments in response to invasive fire ants [33–34], suggesting that this genus may be able to respond rapidly to human-induced environmental change. While there are a only a handful of studies examining the relationship between urbanization and FID in lizards [35–39], one of these reported that urban western fence lizards had significantly reduced FID, consistent with studies in other urban taxa [39]. Thus, there is some evidence of fence lizards being habituated to human presence and/or being subject to low predation risk in general—though it is also possible this reduction in FID is related to other factors, such as proximity of retreat sites. However, little is known regarding how these widespread and successful lizards have changed in other regards, in response to massive urbanization within their range.

Our study focused on urban and rural fence lizards from two replicate populations in Santa Barbara and San Luis Obispo counties. The ability to study response to urbanization in replicate populations effectively geographically isolated from one another, yet similar with regard to both the surrounding native landscape and urban alterations, is critical for discerning whether organismal responses to anthropogenic environmental change are in parallel, and therefore the best explanation for any potential differences that might arise. If animals in replicate urban populations show similar changes, this lends support to the hypothesis that it is urbanization per se, and not some other unmeasured environmental difference between urban/rural sites that the animal is responding to.

Of course, in a natural experiment, it is unlikely for replicate study populations to be identical; however, they should be as similar as possible, as environmental differences within urban habitats may incur different responses [40]. We chose the campuses of Westmont College in Santa Barbara and Cal Poly San Luis Obispo to be our focal urban populations. Westmont and Cal Poly are 95 miles apart and separated by abundant undeveloped land, including mountainous areas in the southern coast ranges. Thus, there exists no complete urban corridor between the two campuses, suggesting that whatever changes may have occurred in lizards on these campuses, they have occurred independently. Both campuses lie in close proximity to undeveloped land, and show a mix of native and introduced vegetation abutting major academic buildings and dormitories, and natural landscaping elements such as sandstone boulders, which are conducive to basking amid vegetation. In addition to buildings, campus landscape alterations include flat open areas such as paths, patios and roads, and more complex elements such as retaining walls and fences, where lizards can also be found (though usually within close proximity to vegetation of some kind). Both campuses have been established for > 75 years (>15 lizard generations), which has been ample time for colonization from nearby rural populations.

We compared campus lizards to rural lizards living in wild lands immediately adjacent to each campus. There was a high possibility of dispersal between each campus and its adjacent rural area, due to their close proximity and lack of significant barriers. Thus, any differences between the two habitat types could represent either a colonization effect, plasticity, or strong, localized selection within campuses that generates small hotspots of urban-adapted lizards. We tested for parallel alterations in antipredator behavior and morphology in campus habitats. We used a series of field-based assays that allowed us to compare behaviors that are associated with an acute antipredator response: aggression in response to a quasi-predation attempt (capture by researcher), escape behavior in three contexts (within a confined area, in response to a predator simulation, and upon release), and sprint performance on a field-portable racetrack. As limb length is often positively associated with sprint speed in lizards [30,41](Losos 1990;, we tested for differences in limb length between campus and rural habitats, as well as for significant associations between limb length and antipredator behaviors. Furthermore, we tested for associations among our different indices of antipredator behavior, to evaluate whether they were independent or might serve as indices of the same underlying functional constraints. We predicted that if predatory release was a major factor affecting campus lizards, they would have decreased aggression, slow escape, reduced response to a predator simulation, reduced sprint speed, and shorter limbs.

## Methods

### Study system

*Sceloporus occidentalis* is a small Phrynosomatid lizard that has a wide distribution across many western states and into northern Baja Mexico [42]. In this study we sampled the coast range fence lizard, *S*. *o*. *bocourtii*. Lizards were captured via noose at Westmont College (34°26'56.33"N, 119°39'39.71"W) and Cal Poly San Luis Obispo (35°18'5.78"N, 120°39'33.84"W; see Introduction for details), and wild lands immediately adjacent to each campus. It was important to sample rural lizards as closely as possible to each campus, so as not to introduce elevational or other differences that might not boil down to human-induced changes per se. Our rural sample was drawn from Poly Canyon (35°18'46.83"N, 120°39'5.86"W), which abuts the Cal Poly campus, and the portion of the Los Padres National Forest (Santa Barbara district) that begins < 2 km from the Westmont campus (34°27'31.53"N, 119°39'33.25"W), with ample vegetated corridors between campus and the National Forest. Both of these rural sites show a mix of native riparian, oak woodland, and chaparral habitats, where lizards are readily found. For the subset of lizards for whom microhabitat was recorded at first sighting, almost 50% of campus individuals were found on pavement or walls, and the other half found on large rocks used in landscaping (N = 59). Only one lizard was spotted in a tree. In contrast, while 16% of rural individuals were spotted on a hiking path, the remainder were on rocks (66%) or in trees (18%) (N = 56).

All applicable international, national, and/or institutional guidelines for the care and use of animals were followed. Lizards were captured with permission from the California Department of Fish and Wildlife. All methods were approved under by the Institutional Review Board (IRB) at Westmont College (protocol #855). All animals were released without any apparent harm at the point of capture.

For each lizard, we measured snout-vent length (SVL), body weight, femur, humerus, radius/ulna, and tibia/fibula lengths. Femur and humerus measurements were made with the limb held out at a 90° angle from the abdomen, beginning with the abdominal wall where the limb inserted and ending at the most distal point where the limb bends. Radius/ulna and tibia/fibula measurements were made from the most proximal point at the bend of the limb to the beginning of the forefoot/hindfoot regions. All measurements used in the morphological analysis were performed in a consistent manner by the lead author. We sexed individuals by checking for enlarged post-anal scales, present only on males [42].

### Behavior assays

All behavioral assays were conducted in the field within 10 minutes of capture, in order to effectively examine acute antipredator response in the wild. We examined antipredator behavior in campus and rural lizards via five different assays: (1) biting in response to capture (2) confined escape behavior in an open field test, (3) response to predator simulation in an open field test (4) escape latency upon release, and (5) sprint behavior. We were able to correlate behaviors within individuals for assays (1)-(4), as these were performed on the same sample of individuals in 2015 for both Santa Barbara (Campus: Males N = 19; Females N = 24; Rural: Males N = 31; Females N = 26) and San Luis Obispo (Campus: Males N = 11; Females N = 12; Rural: Females N = 9; Males N = 6) counties. Sprint behavior was assayed for another sample in 2016, involving only Santa Barbara county (Males: N = 11; Females: N = 11).

To assess aggression, we employed biting in response to capture as a binary response variable, defined as "yes" or "no" depending on whether they opened up their mouths in an attempt to bite when being extracted from the noose. Confined escape behavior and response to predator simulation were assayed in an open field test constituted by placement within a flat-bottomed, circular behavioural arena (3.8 L, 26 cm diameter), which was sterilized between each trial with ethanol. Such artificial open-field tests have been used to assess reptile behaviours in various contexts [43–45], and that animals can are able to move freely in small, artificial enclosures, and even exhibit mating behaviours [46]. Body temperature of each lizard was measured via cloacal thermometer subsequent to their trial (DeltaTrak model 11063, Pleasonton, CA). To our surprise, while some lizards moved rapidly and agitatedly upon being deposited in the arena, scrambling against the sides, others exhibited tonic immobility (otherwise known as playing dead, or thanatosis) [47]. In this case, they did not move at all upon placement in the arena, and often remained in awkward and unnatural poses. Thus, confined escape behavior was considered as a binary response variable, with two major categories of response: (a) tonic immobility or (b) active movement. The predator simulation, modeled after previous studies that have successfully used sticks or brushes to stimulate antipredator behaviour in lizards [48–50] was performed one minute after the lizard was placed in the arena, and consisted of five rapid prods of a fishing rod (same as used for noosing the lizards) in the interior of the arena, without making contact with the lizard. Some lizards that exhibited tonic immobility upon placement in the arena continued to be inert in spite of the prod, while others did in fact spring into action in response to the predator simulation; initially active lizards also showed a spike in rapid movement in response to the simulation. Thus, there continued to be two well-delineated categories of response to the simulation: (a) delayed response—no response, or no response until the fourth or fifth prod, and (b) rapid response—immediate, rapid movement at the first prod.

We also assayed latency to escape upon release subsequent to the trial in the behavioural arena. The researcher releasing the animal stood with their feet together, and placed the animal upon the ground in front of them. If the animal did not sprint away immediately, the researcher took a step back. If the animal still did not move, the person leaned forward. If the animal still did not move, the person reached down and touched the lizard. We categorized individual lizard responses into two main categories: (a) rapid escape, if the lizard sprinted immediately or as the researcher stepped back, or (b) delayed escape, if the animal did not flee until the researcher leaned over them, or touched them.

In a separate subset of lizards, sprint behavior was assayed using a 48 inch field-portable racetrack, with wooden base, sandpaper-lined track for traction, a clear plexiglass cover, and velcro-secured entry/exit points on either end. Lizards were gently placed on the track at one end, and trials were videorecorded using an iPhone (version 6S Plus) placed on a tripod. While some lizards immediately sped to the other side of the track, others stopped intermittently along the track. Each time a lizard stopped, it was gently prodded with the noosing rod from behind, encouraging it to run farther. The body temperature of each lizard was measured after the sprint trial via cloacal thermometer. Videos were coded for relevant behaviors using Solomon Coder (András Péter 2006–2014). We looked at three aspects of sprinting behavior on the track: total time taken to traverse the track, average speed while moving, and number of stops made on the track. Average speed while moving was calculated by dividing the total track length by the amount of time spent running on the track (i.e., not including the time spent still).

### Statistical analyses

Biting upon capture, confined escape behavior, response to predator simulation, and escape latency were all analyzed with logistic regression, with SVL and body temperature as covariates, and sex, county and habitat type as main effects, as well as a two-way interaction between habitat and sex. Relationships among all four of these behavioural variables were analyzed using Pearson’s χ^2^ analyses. Continuous sprint behaviors (time on track, sprint speed) were natural log-transformed to meet assumptions of normality, and analyzed using ANCOVA with SVL and body temperature as covariates, sex and habitat type as main effects. Since number of stops involved count data, it was analyzed using a generalized linear model with Poisson distribution and log link function, with SVL and body temperature as covariates, sex and habitat type as main effects. No sex by habitat interaction was included in sprint analyses, as we had lower power to detect such an interaction.

To account for differences in limb size with body size, relative limb measurements were calculated by dividing by each measurement by an individual’s SVL. Limb measurements were analyzed using an ANCOVA and sex, county, and habitat type as main effects, and the two-way interactions between habitat and sex. Since femur length was most well differentiated between the habitat types (see Results), we also tested for a relationship between relative femur length and all five behaviors.

We included all relevant independent variables in our starting model for each of the dependent variables listed above, and then proceeded to exclude all variables with *P* > 0.2. All analyses were conducted with JMP 10.0.0 (SAS Institute Inc., Cary, NC).

## Results

### Behavior

**Fig 1:**
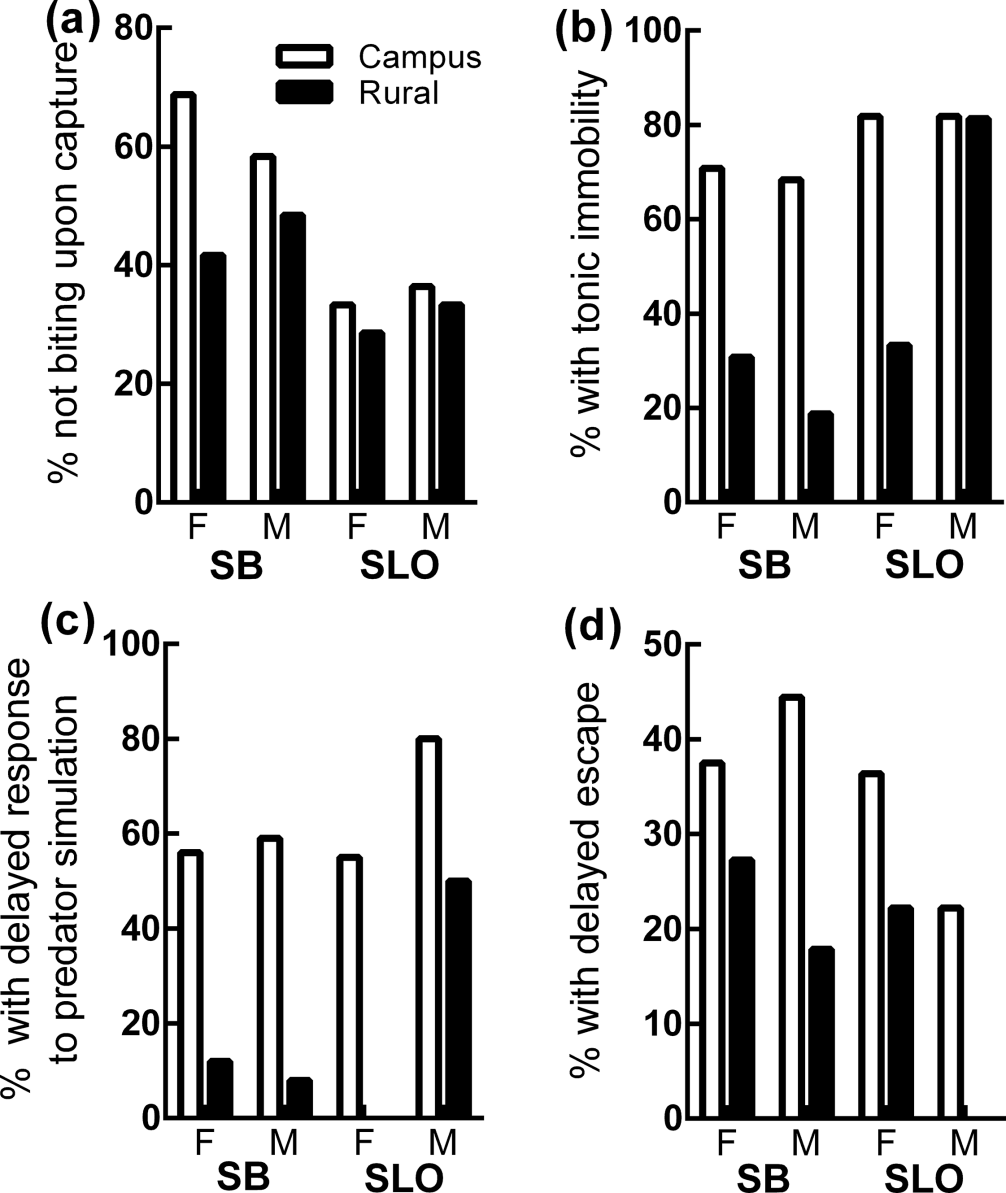
Proportion of individuals exhibiting different categories of antipredator behaviour in campus and rural *S*. *occidentalis*, presented by county (SB: Santa Barbara; SLO: San Luis Obispo) and sex (Female/Male).

Results from behavioral analyses are summarized in Table 1 and Fig 1. The final model for biting at capture contained only county and SVL. Santa Barbara lizards were significantly more likely to bite than San Luis Obispo lizards, and larger lizards were marginally significantly more likely to bite than smaller lizards.

**Table 1:** Results of likelihood ratio test from logistic regression of four antipredator behaviors exhibited by *S*. *occidentalis* upon capture (biting), upon placement in a arena (confined escape behavior and response to predator stimulus), and upon release (escape latency). Confidence intervals (CI) are provided for each odds ratio.

	*χ2*	*df*	*P*	Odds ratio	Lower 95% CI	Upper 95% CI
**Biting upon capture:** N/Y						
County (SB vs. SLO)	5.09	1.00	0.02	2.65	1.13	6.55
SVL	4.19	1.00	0.04	0.13	0.02	0.92
**Confined escape behaviour:** tonic immobility/active movement						
Habitat Type (rural vs. campus)	17.17	1.00	< .0001	6.25	2.57	16.27
County (SB vs. SLO)	2.05	1.00	0.15	2.01	0.18	1.29
Body Temperature	3.99	1.00	0.04	1.17	1.02	1.39
**Response to predator simulation:** active movement/little or no movement						
Habitat Type (rural vs. campus)	22.60	1	< .0001	9.84	3.64	30.37
Sex (Male vs. Female)	2.83	1	0.09	0.44	0.16	1.15
Body Temperature	2.68	1	0.10	5.70	0.71	50.55
**Escape latency:** delayed/rapid						
Habitat Type (campus vs. rural)	4.06	1	0.04	2.79	1.06	8.07
Body Temperature	2.69	1	0.10	0.20	0.03	1.35

The final model for confined escape behavior contained habitat type, county and temperature. The difference between counties was not significant, but a significantly higher proportion of campus lizards showed tonic immobility than rural lizards when placed in the arena (Fig 1B). Those that were warmer were also more likely to exhibit movement. The campus/rural difference in tonic immobility could not be explained by mean differences in body temperature, however, as mean temperature was nearly the same for both habitat types (campus: 26.6°C ± 2.93; rural: 26.8°C ± 3.04). Fig 1B suggests that the relationship between tonic immobility and habitat type is primarily driven by both males and females in Santa Barbara and females in San Luis Obispo. There was no apparent difference, however, between rural and campus males in San Luis Obispo—in this case, most were immobile in both habitat types.

The final model for the predator simulation contained habitat type, temperature, and sex. A significant habitat type effect revealed that in addition to more often exhibiting tonic immobility upon placement in the arena, campus lizards more frequently showed delayed responses to the predator simulation (Fig 1C). Temperature (*P =* 0.010) *and* sex (*P =* 0.093) did not significantly affect responses to the simulation, though there was a trend for warmer/female lizards to show rapid responses. The final model for escape latency contained habitat type and temperature. Campus lizards showed delayed escape upon release (Fig 1D). There was no significant relationship between temperature and escape latency.

Those lizards that bit at capture were not more likely to respond to the predator simulation (χ2 = 0.00; *P* = 0.98), nor were they more likely to escape rapidly (χ2 = 0.31; *P* = 0.58). There was no relationship between confined escape behavior and biting at capture (χ2 = 1.06; *P* = 0.30); that is, those that exhibited tonic immobility upon placement in the arena were not less likely to bite. Those that showed tonic immobility upon placement in the arena, however, were less likely to escape rapidly (χ2 = 5.39; *P* = 0.02). Individuals with tonic immobility were also more likely to have a delayed response to a predator simulation (χ2 = 16.91; *P* < 0.0001). Finally, there was a marginally significant relationship between response to predator simulation and escape latency (χ2 = 3.12; *P* = 0.08), where lizards that responded rapidly to the predator simulation were also faster to escape.

**Fig 2:**
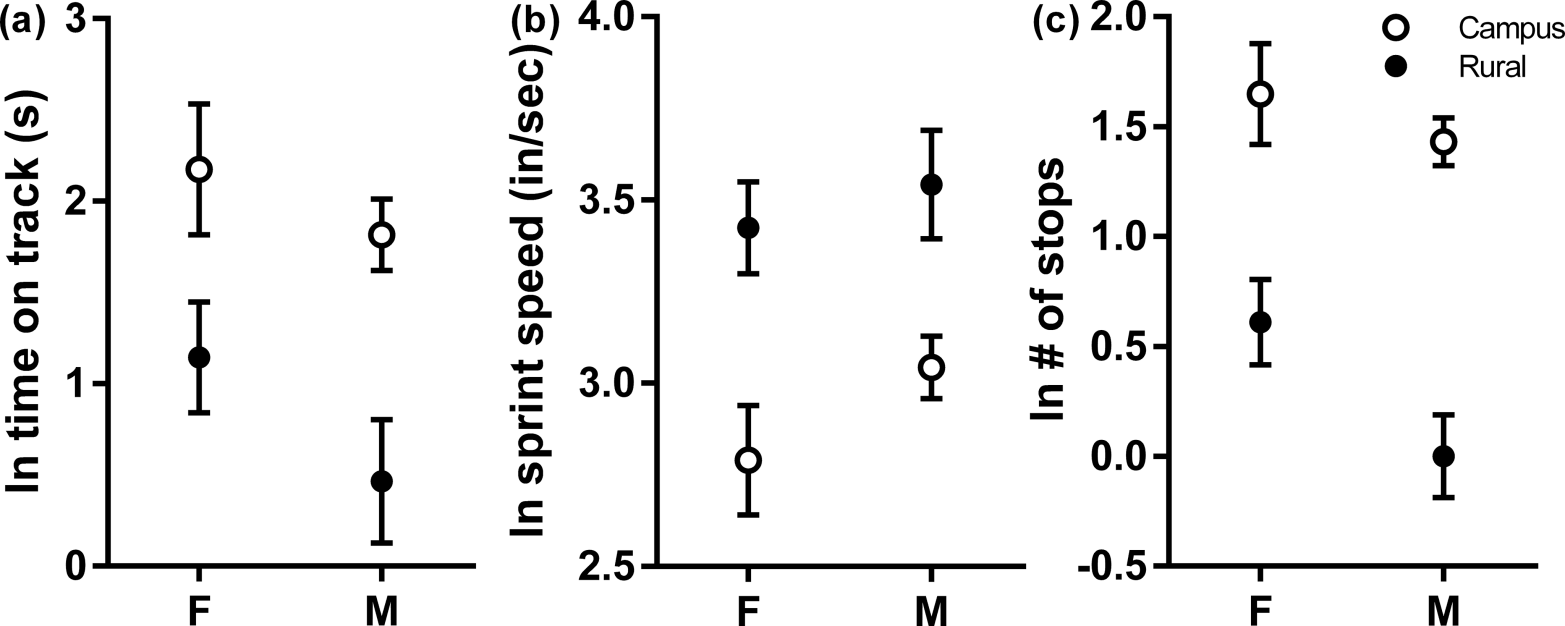
Least square means of sprint behaviors on a field-portable race-track in campus and rural *S*.*occidentalis* by sex (Female/Male). Standard errors of the means are shown.

Sprint performance on the field-portable racetrack showed strong differentiation between campus and rural habitats, and habitat type was the only variable in the final model for all three dependent variables. (Fig 2). Campus lizards took significantly longer to traverse the track (F_1,22_ = 11.48; *P* = 0.003; Fig 2A), which appears to have been both a function of lower sprints speeds and a higher number of stops. Campus lizards also had significantly lower sprint speeds (F_1,22_ = 16.46; *P* = 0.005; Fig 2B) and stopped more (χ^2^ = 7.22; *P* = 0.007; Fig 2C).

### Morphology

**Fig 3:**
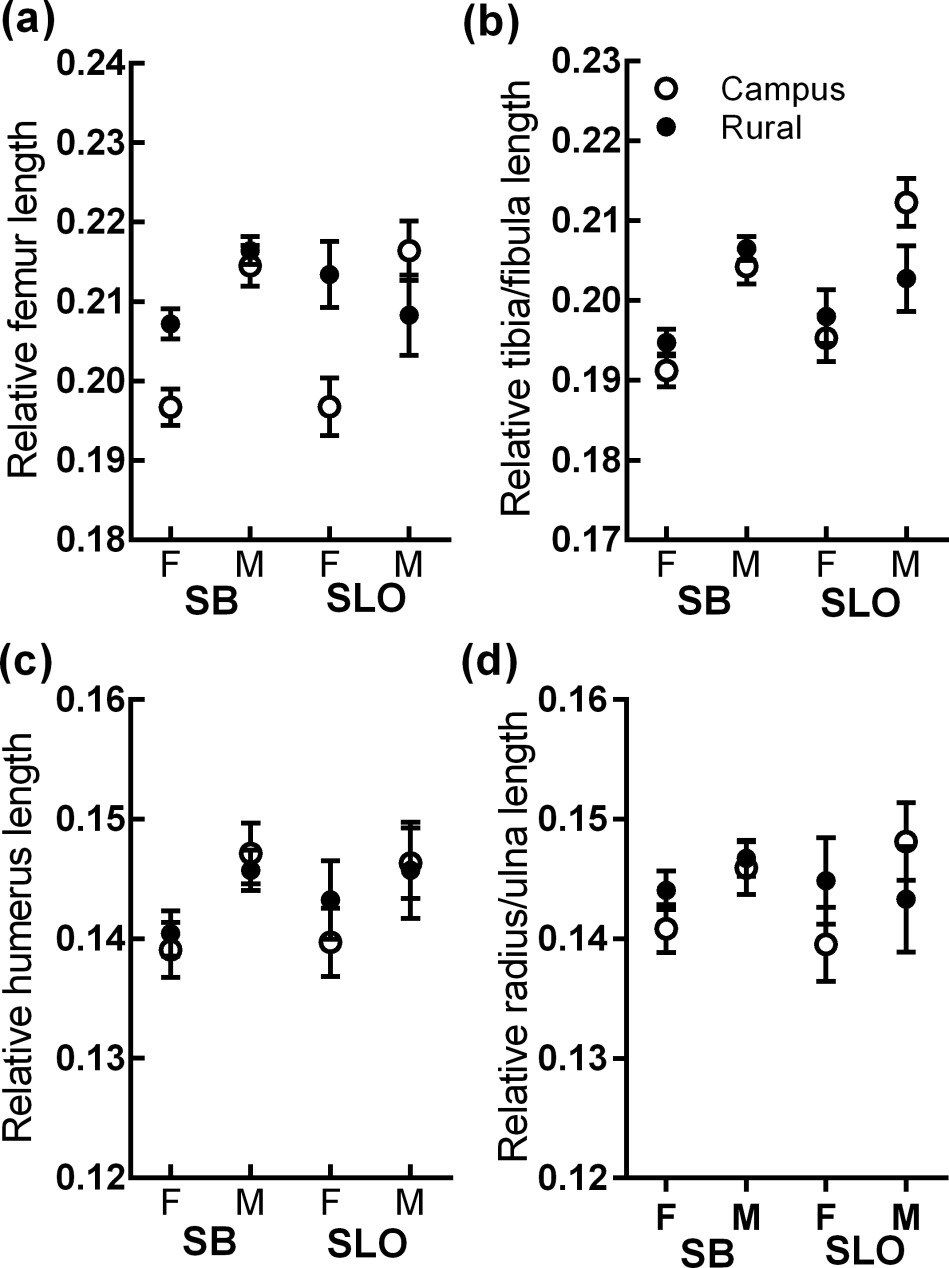
Model-adjusted least square means of relative limb measurements in campus and rural *S*. *occidentalis*, presented by county (SB: Santa Barbara; SLO: San Luis Obispo) and sex (Female/Male).

The results of analyses for morphological variables are provided in Table 2 and Fig 3. For all four limb measurements, sex was a significant main effect in each model, with males always being larger than females. The final model for relative femur length contained sex, habitat type, and the interaction between the two. The significant sex x habitat type interaction revealed that relative femur length was significantly shorter in campus females (Fig 3A). The final model for tibia/fibula length contained both sex and county, with marginally significantly longer tibia/fibulas in San Luis Obispo. The final model for relative humerus length, and radius/ulna length contained only sex.

**Table 2:** Results from ANCOVA of relative limb measurements of *S*. *occidentalis* used in behavioral trials.

	*Df*	*F*	*P*
**Femur**			
Habitat Type	1,122	9.46	0.003
Sex	1,122	42.30	< .0001
Habitat Type*Sex	1,122	10.31	0.002
**Tibia/Fibula**			
Sex	1,123	59.81	< .0001
County	1,123	3.13	0.080
**Humerus**			
Sex	1,124	12.01	0.001
**Radius/Ulna**			
Sex	1,124	6.14	0.014

Relative femur length was not significantly related to biting upon capture (χ2 = 0.45; *P* = 0.50), escape latency (χ2 = 0.10; *P* = 0.74), or sprint speed (F_1,22_ = 0.08; *P* = 0.76). While there was no overall relationship between femur length and confined escape behavior, note that if the analysis was conducted by habitat type there was a marginally significant trend within campus females, such that shorter-legged females were more likely to exhibit tonic immobility (χ2 = 3.22; *P* = 0.07) and delayed antipredator response (χ2 = 3.47; *P* = 0.06) than longer-legged females. Note that similar analyses were conducted with the other three limb measurements, but none were significantly related to any behavioral variables (results not presented here).

Differences in campus and rural lizards resulting from the morphological and behavioral analyses described above are summarized succinctly in Table 3.

**Table 3:** Summary of differences between *S*.*occidentalis* living in campus and rural habitat types in Santa Barbara (SB) and San Luis Obispo (SLO) counties. Instances where no significant differences occur between campus and rural habitat types are indicated by a "~".

		Campus	Rural
***Behaviour***			
	Biting at capture	~	~
	Confined escape	tonic immobility	rapid movement
	Predator simulation	delayed response	rapid response
	Escape latency	delayed escape	rapid escape
	Track time	long	short
	Sprint speed	slow	fast
	Number of stops	many	few/none
***Morphology***			
	Femur length	shorter (females only)	longer (females only)
	Tibia/fibula length	~	~
	Humerus length	~	~
	Radius/ulna length	~	~

## Discussion

We found compelling evidence of parallel differentiation in behavior and morphology between campus and rural fence lizards in Santa Barbara and San Luis Obispo, California. On college campuses, lizards exhibited behaviors that could be associated with delayed escape from predators, such as an increase in tonic immobility, delayed response to a predator simulation, delayed escape upon release, and reduced sprint performance. Females on both campuses also had shorter femurs relative to their body size. Together, these findings are consistent with predictions generated by the hypothesis that there has been a reduction of predation pressure resulting in less developed antipredator defenses, though changes in predator communities and habituation to human presence may also play a role.

### Behavior assays

We encountered a surprisingly stark contrast in behavior among lizards placed in the arena for confined escape behavior trials, with some individuals showing tonic immobility, and others scrambling rapidly around the arena. This tonic immobility was particularly likely to persist for campus lizards through the predator simulation, and even upon release, with some lizards not even attempting to escape when nudged. Tonic immobility has been observed in species throughout the animal lineage, and is considered an unlearned reflexive response elicited by the perception of mortal danger during physical restraint [47]. There are several hypotheses regarding the function of tonic immobility, with some interpreting it as a maladaptive fear response, and others suggesting it may increase changes of escape by distracting predators [47,51]. In campus fence lizards, a greater propensity for tonic immobility may represent (1) an effective way to escape humans, (2) an effective way to escape campus predators, or (3) a maladaptive response due to limited exposure to predators, causing handling to be a more terrifying experience in general. The first explanation (1) seems unlikely, as to our knowledge there are not large numbers of humans physically restraining lizards on these campuses; and even if this were the case, it is unclear why those that exhibit tonic immobility would be more likely to escape than those that run away quickly upon being put down. The second explanation (2), suggesting that tonic immobility is an effective way to escape predators, might be more reasonable, but would require a greater understanding of campus predator community composition, and how numbers differ from rural populations. It is possible that tonic immobility may be more effective for predators that are inclined to drop prey before consuming it, lose interest in prey if it is immobile, or have difficulty finding it again due to enhanced crypsis [47, 51]. Tonic immobility has been shown to last longer in urban than in rural birds, and there is evidence that this behaviour is associated with higher predation by cats [26]. At our rural sites, there are a range of potential predators including numerous raptors (e.g., red-tailed hawks, red-shouldered hawks, Cooper’s hawks, sharp-tailed hawks and kestrels), snakes (gopher snakes, kingsnakes, and rattlesnakes), and generalist mammals (e.g., bobcats, coyotes, foxes). Potential predators on campus include more typical urban species such as feral cats, raccoons, skunks, jays, and occasional hawks. However, these two campuses in particular do not appear to have large feral cat populations, and raccoons and skunks are not commonly seen during the daylight hours when lizards are most active. And while avian predation may certainly occur in campus habitats, we hypothesize that predation rate is indeed reduced, as shown in studies of predation in other urban environments [11,26,27]. Nevertheless, it is still possible that the increased frequency of tonic immobility in campus lizards is a response to differences in predator communities, regardless of the predation rate, and increases the likelihood of escape from campus predators.

The third explanation (3), which suggests that tonic immobility is a maladaptive response due to more limited exposure to predators, is the most straightforward. Studies in wild birds have suggested that a longer time spent in tonic immobility is associated with decreased ability to escape predators [52,53], suggesting that higher frequency of tonic ability could be a result of predatory release in campus lizards. Given that tonic immobility is considered an unlearned response, at first glance an increase in frequency might seem to be evidence of evolutionary change. However, it is possible that there have been plastic physiological alterations in campus lizards that increase the chances of tonic immobility in response to physical restraint. Animals can become habituated to stimuli that originally promoted tonic immobility, and thus rural lizards may overcome this innate response to physical contact with a predator over development [51](though how likely it is that lizards would repeatedly survive such encounters is another matter). It may be that in the absence of frequent predatory attacks on a regular basis (especially given the high density but low threat of humans, as the predominant large mammal on campus), such an intense experience as being held by a researcher is more of a fear-engendering event than it would be for rural lizards.

While our results are consistent with a scenario of predatory release, it is also possible that campus lizards may be so accustomed to human presence that being handled by a human is more likely to send them in to a state of shock than rural lizards, who may be more likely to classify humans as a threat in the first place. This would imply an advanced ability in *S*. *occidentalis* to differentiate between different predator types, which would certainly be of interest to investigate further.

While we did find a higher proportion of individuals with tonic immobility and delayed response to predator simulation in females from both campuses, and in males from Westmont, campus and rural habitat types in San Luis Obispo exhibited a similar (and high) proportion of males exhibiting tonic immobility. Note, however, that rural lizards were still more likely to snap into an active response during the predator simulation, which is consistent with an enhanced antipredator ability. Future work should focus on measuring time to movement (without the interruption of a predator simulation), as in recent bird studies [26], as ability to escape predation in some rural populations may be a function not only of whether an individual exhibits tonic immobility, but how long it lasts.

We also found evidence that our indices of antipredator behavior were related in some cases, but not in all. There was no relationship between biting and either the confined escape or predator simulation assays, suggesting that the immediate aggressive response upon capture was independent of both of these two indices. There was a relationship, however, between biting and escape latency, with those that did not bite being more rapid at escape. Furthermore, our confined escape behavior and escape latency were also significantly related, suggesting that these may be estimating the same underlying behavioral trait, at least in part.

### Sprint performance

Sprint performance was markedly different between the two habitat types in Santa Barbara. We observed that as soon as a rural lizard was placed on the track, it sped to the other side, often without pause (median = 0 stops; range 0–3 stops). Campus lizards, on the other hand, exhibited more of a stop and go pattern (median = 4 stops; range 2–7 stops), and generally needed encouragement with the prod to continue down the track. Furthermore, not only did they differ in sprint pattern, but they also differed in sprint speed, with rural lizards running more than 1.5 times as fast (mean campus speed = 20.5 in/sec; mean rural speed = 33 in/sec). These findings match reports in other lizard populations, where lizard populations that are thought to have higher levels of predation are typically faster [54–56]. Thus, this reduction in sprint performance on campus is more likely to reflect predatory release than differences in predator communities, as lower sprint speeds in general are unlikely to be advantageous for evading either the rural or urban predators potentially present in these habitats. Predatory release for campus lizards could result in relaxed selection on speed, or simply less developed limb musculature due to less intensive use. It may also reflect habituation to human presence, as many urban taxa show reduced FIDs in response to human approach [9], or differences in distance to refugia in campus and rural habitats. Several studies show that FID increases with increasing distance to refuge in diverse taxa [36,57–59] and one study in garden skinks has found both faster sprint speeds and farther distances to refugia in urban lizards [60]. Future work should compare distances to refugia in campus and rural habitats, to determine whether this could be major factor affecting sprint performance.

While high tonic immobility, delayed escape latency, and slower sprint performance are all consistent with our predictions for a scenario of predatory release is important to note that these are all behaviors that are being performed in the context of acute threat i.e., capture by a researcher. It is possible that if boldness and aggression are uncoupled in acute vs. chronic conditions, these animals may yet show themselves as more bold, aggressive, and even exploratory in other contexts. For instance, they may still be more bold when approached by humans [39], and generally engage in more bold, exploratory foraging on campus (due to habituation to humans, increased resource availability, shorter distances to refugia, and/or reduced predatory threat) than they would in rural habitats. Furthermore, even in our study, while reduced sprint speed in campus lizards is consistent with predatory release, it is not necessarily inconsistent with boldness. Slower, intermittent sprint patterns can reflect boldness (or habituation) in human presence, and/or boldness in a low-predation environment.

### Morphology

Our finding that campus females have shorter femurs is opposite in direction to a recent studies in urban *Anolis sagrei* and *A*. *cristatellus* which found evidence of longer hindlimb span in urban males [21,61] (females not studied), but parallels findings in *Anolis carolinensis* on a university campus [35]. Yet another study in garden skinks, *Lampropholis guichenoti* found that both male and female lizards in urbanized habitats had significantly faster sprint speeds, but this was not explained by differences in limb length [60]. Combined with the present study, these previous lizard studies, as well as work showing changes in lizard morphology and behavior in response to species invasions [33,62] (but see [63]) serve to highlight both the intriguing idea that diverse lizards may be undergoing morphological change in response to urbanization on a worldwide scale, and also that responses to urban environments may differ by urban characteristics and/or by species.

Detailed study of *Anolis* lizard morphology suggests shorter-limbed individuals are slower, especially on broad surfaces [41,64]. A general positive correlation between sprint speed and limb length has also been reported in *S*. *occidentalis*, though populations with longer limbs on average appear to be more effective on arboreal than on broad surfaces [30]. Previous work in *Sceloporus* has also suggested that males are faster on average than females [56,65]. Nevertheless, femur length was not related to sprint speed, either within or across habitats, and thus did not satisfactorily address why females might have developed shorter limbs. It is possible that the lack of a significant association between limb length and speed in our study may be a consequence of a low power, as the study that did find such a relationship had >100 *S*. *occidentalis* [30](though note that a *Sceloporus merriami* study found no relationship—see [56]), whereas our sprint performance sample size was limited to 22 individuals. However, it remains the case that campus males, who did not have shorter limbs on average, were still slower than rural males. The fact that we had sufficient sample size to demonstrate dramatic differences in sprint performance between habitats, suggests that relative limb length may be only one of several key variables determining sprint performance. It will be interesting to examine relevant physiological factors, such as anaerobic metabolism, which may favor more rapid burst speeds [66]. Whatever the mechanism, rural lizards excelled campus lizards on a flat racetrack, in spite of the fact that campus lizards spend much of their time on flat surfaces, such as paved walkways. This suggests that not only may limb length not be paramount, but substrate type may also be relatively unimportant in generating differences in sprint performance in this system.

Interestingly, there was some evidence that for campus females, those with shorter limbs had a greater tendency to show tonic immobility, and were less likely to respond to the predator simulation. Thus, there may be inter-individual differences in behavioral strategy based on limb lengths where longer-limbed females (but not males) are more likely to try a rapid escape strategy. If sprint speed per se is not the factor upon which this decision hinges, it may be that shorter limbs provide other advantages in navigating urban habitats for females. It may be that the two sexes have differentiated in habitat use in campus environments, due to differences in territorial behavior and/or reproductive requirements as females may prefer to spend at least part of the time in optimal egg-laying areas. Future work should examine habitat use in more detail to illuminate this possibility more, and how effective locomotion in different areas might be better served by different limb lengths.

While it cannot explain why shorter-limbed females might be more likely to show tonic immobility, another potential explanation for shorter limbs in campus females in general is potential selection during egg-laying. *Sceloporus* females use both fore and hind limbs to dig holes in which to deposit their eggs. Campus soils tend to be irrigated and enriched due to landscaping efforts, and may serve as a softer substrate for digging than typically dry, rocky soils in rural areas. Thus, if limb length is important for hole digging, it may be subject to relaxed selection in the campus environment.

Limb development has been shown to exhibit plasticity and be influenced by habitat structure in *Anolis* lizards [67,68], and *S*. *occidentalis* exhibits plasticity in growth rates under different temperatures regimes [69,70]. However, hind limb plasticity in *S*. *occidentalis* with respect to incubation temperature disappears with age, and hind limb length appears more heritable and less plastic than other morphological traits, including fore limb length [71,72]. Thus it is possible that there has been evolutionary change in female lizards on these two campuses, if selection for short limbs (or relaxation of selection on long limbs) is strong enough to overcome gene flow from surrounding rural habitats.

### Conclusion

We found marked evidence of instances of parallel behavioral and morphological differentiation in campus lizards in both Santa Barbara and San Luis Obispo, all of which are consistent with a change in predator communities and/or relaxation of predation pressure, though habituation to human presence may also play a major role. It is highly likely that there is dispersal between each campus and its adjoining rural populations, thus the behavioral and morphological differences we observed may be developmentally plastic rather than genetically canalized. In fact, the vast majority of phenotypic change in human-altered environments appear to result from plasticity (Hendry et al. 2008). However, the degree to which antipredator behavior and morphology represents a plastic or evolutionary response to urbanization commands more thorough investigation. We anticipate that further inquiry into additional behavioral, morphological, physiological, genetic and life-history variables in a wider variety of urban habitats across the western fence lizard range will contribute much to our understanding of how species manage to colonize and thrive in human-altered environments.
